# Heat-Killed *Lacticaseibacillus paracasei* Repairs Lipopolysaccharide-Induced Intestinal Epithelial Barrier Damage via MLCK/MLC Pathway Activation

**DOI:** 10.3390/nu15071758

**Published:** 2023-04-04

**Authors:** Zhixin Xie, Gongsheng Zhang, Rongxu Liu, Yucong Wang, Anna N. Tsapieva, Lili Zhang, Jianchun Han

**Affiliations:** 1 College of Food Science, Northeast Agricultural University, Harbin 150030, China; 2 Heilongjiang Green Food Science Research Institute, Harbin 150030, China; 3 Department of Molecular Microbiology, FSBSI Institute of Experimental Medicine, Acad.,197376 St. Petersburg, Russia

**Keywords:** heat-killed *Lacticaseibacillus paracasei*, probiotics, intestinal epithelial barrier, tight junction, inflammation

## Abstract

Intestinal epithelial barrier function is closely associated with the development of many intestinal diseases. Heat-killed *Lacticaseibacillus paracasei* (HK-LP) has been shown to improve intestinal health and enhance immunity. However, the function of HK-LP in the intestinal barrier is still unclear. This study characterized the inflammatory effects of seven HK-LP (1 μg/mL) on the intestinal barrier using lipopolysaccharide (LPS) (100 μg/mL)-induced Caco-2 cells. In this study, HK-LP 6105, 6115, and 6235 were selected, and their effects on the modulation of inflammatory factors and tight junction protein expression (claudin-1, zona occludens-1, and occludin) were compared. The effect of different cultivation times (18 and 48 h) was investigated in response to LPS-induced intestinal epithelial barrier dysfunction. Our results showed that HK-LP 6105, 6115, and 6235 improved LPS-induced intestinal barrier permeability reduction and transepithelial resistance. Furthermore, HK-LP 6105, 6115, and 6235 inhibited the pro-inflammatory factors (TNF-α, IL-1β, IL-6) and increased the expression of the anti-inflammatory factors (IL-4, IL-10, and TGF-β). HK-LP 6105, 6115, and 6235 ameliorated the inflammatory response. It inhibited the nuclear factor kappa B (NF-κB) signaling pathway-mediated myosin light chain (MLC)/MLC kinase signaling pathway by downregulating the Toll-like receptor 4 (TLR4)/NF-κB pathway. Thus, the results suggest that HK-LP 6150, 6115, and 6235 may improve intestinal health by regulating inflammation and TJ proteins. Postbiotics produced by these strains exhibit anti-inflammatory properties that can protect the intestinal barrier.

## 1. Introduction

More than 10 million people worldwide are affected by ulcerative colitis and Crohn’s disease [1]. The human intestine has a complex microenvironment, and the intestinal barrier contributes to the preservation of the intestinal anatomy [2]. It has been demonstrated that intestinal flora dysbiosis and intestinal epithelial barrier dysfunction are directly related to the development of UC and CD [3]. When the intestinal barrier is compromised, the local immunity is activated, and the tight junction (TJ) protein structure is altered, causing cytokine imbalance and mediating a decrease in intestinal permeability [4,5,6,7]. TJ proteins, formed by cell-to-cell interactions, compose a major defense barrier in the intestinal epithelium. Simultaneously, myosin light-chain kinase (MLCK) interacts with the TJ protein to regulate epithelial cell permeability and maintain the integrity of intestinal function [8]. MLCK mediates myosin light-chain (MLC) phosphorylation (p-MLC), leading to the disruption of TJ proteins and increased intestinal epithelial cell permeability [9].

A previous study showed intestinal barrier injury could cause inflammation and gut hypermotility, which can lead to the onset of inflammatory bowel disease [10]. Therefore, it is vital to maintain the integrity of the intestinal barrier. In vitro studies have shown that *Lactiplantibacillus plantarum* MB452 reduces intestinal epithelial cell permeability and improves the expression and distribution of Zona Occludens (ZO-1) [11]. The animal experiment showed that *L. plantarum* L15 is able to maintain the integrity of the intestinal epithelial barrier by preventing the production of genes associated with the NF-κB signaling pathway [12]. 

Probiotics are live microorganisms, and when sufficient amounts of probiotics are consumed, certain changes in intestinal health occur, which in turn provide benefits to the organism [13]. These benefits can effectively improve mild bowel diseases such as ulcerative colitis [14]. However, the limitations of probiotics, such as intolerance to oxygen, heat, and storage, have led researchers to focus on the probiotic effects of inactive microorganisms and their metabolites [15]. In 2021, a consensus statement was officially published in Nature Reviews Gastroenterology and Hepatology, identifying postbiotics as “preparations of inactivated microorganisms and/or their components, including inanimate organisms, bacterial metabolites, and/or post-cleavage components that are beneficial to host health”. Heat-killed *L. paracasei* effectively ameliorates the intestinal inflammatory response in the intestine and maintains the intestinal barrier [16]. It was shown that neither live nor heat-killed *Bifidobacterium animalis* subsp. *Lactis* BB-12 (BB-12) and *L. rhamnosus* GG (LGG) directly induced inflammatory responses in the epithelium. However, pre-treatment with heat-killed BB-12 and LGG reduced the responses induced by inflammatory stimuli [17].

Mounting evidence suggests that *L. paracasei* ameliorates inflammation during intestinal injury [18,19]. Furthermore, *L. paracasei* 1602 prevents pro-inflammatory cytokine production induced by Th1 cells [20]. Moreover, *Lactiplantibacillus paracasei* subsp. *paracasei* NTU 101 significantly downregulated the expression of inflammatory cytokines such as TNF-α, IL-6, IFN-γ, and IL-12 in animal experiments [21]. Additional study has shown that *L. paracasei* MCC1849 can potentially increase resistance to the common cold (influenza) in susceptible individuals, prevent decreased immune strength and fatigue, and improve the immune system in the elderly [22].

Previous research has demonstrated that HK-LP ameliorates intestinal epithelial cell barrier dysfunction [23]. However, the effects of HK-LP from different cultivation time points on lipopolysaccharide (LPS)-induced barrier dysfunction in the human intestinal epithelium has not been reported. Therefore, the effects of HK-LP at different cultivation times on the LPS-induced Caco-2 monolayer cell model were assessed. Furthermore, we investigated the effects of *L. paracasei* 6105, 6110, and 6235 because these strains are useful and widely used in the food industry. Next, we investigated how HK-LP affected Caco-2 cell monolayers in culture, focusing on intestinal barrier damage and inflammatory mediators.

## 2. Materials and Methods

### 2.1. Reagents and Materials

The Caco-2 human colon carcinoma cell line was obtained from Procell Life Science & Technology Co., Ltd. (Wuhan, China). LPS and fluorescein isothiocyanate (FITC)-dextran (4 kDa) were purchased from Sigma (St Louis, MO, USA). All other cell culture materials were purchased from VivaCell Bioscience (Shanghai, China), including Fetal bovine serum (FBS), Dulbecco’s modified Eagle’s medium (DMEM), penicillin–streptomycin, trypsin ethylenediaminetetraacetic acid (EDTA; without phenol red), Hanks’ balanced salt solution (HBSS), and phosphate-buffered saline (PBS). Antibodies for β-actin (66009-1-lg), MyD88 (23230-1-AP), TLR4 (19811-1-AP), Claudin-1 (13050-1-AP), Occludin (27260-1-AP), ZO-1 (21773-1-AP), NF-κBp65 (10745-1-AP), MLCK (21642-1-AP), MLC (10906-1-AP), horseradish peroxidase (HRP)-conjugated Affinipre Goat Anti-Mouse IgG (SA00001-1), HRP-conjugated Affinipre Goat Anti-Rabbit IgG (SA00001-2), CoraLite488-conjugated Af-finipre Goat Anti-Rabbit IgG (SA00013-2), and CoraLite594-conjugated Affinipre Goat An-ti-Rabbit IgG (SA00013-4) were obtained from the Proteintech Group, Inc. (Wuhan, China); p-MLC (Thr18/Ser19) (3674), and phospho-p65 (Ser536) (3033) were purchased from Cell Signaling Technology (Danvers, MA, USA).

### 2.2. Bacterium Cultures and Preparation

A total of seven *L. paracasei* strains were obtained from the China Center of Industrial Culture Collection (CICC, Beijing, China). *L. paracasei* 6105 was isolated from a human cavity; *L. paracasei* 6110 was isolated from the human intestinal tract; *L. paracasei* 6115 was isolated from human tooth decay; *L. paracasei* 6235 was isolated from naturally fermented yak; *L. paracasei* 20252 was isolated from kefir grains; *L. paracasei* 22732 was isolated from the fermentation pit; and *L. paracasei* 24825 was isolated from white sour soup. To culture these strains, a Man, Rogosa, and Sharpe (MRS) broth (AoBox, Beijing, China) was used. The cultures were then aerobically incubated at 37 °C for 24 h. Each culture underwent two successive 24 h transfers in their respective broths.

*L. paracasei* strains were cultured in the MRS broth for 18 h and 48 h, respectively. The cultures were then centrifugated at 8000× *g* for 20 min at 4 °C to separate the bacterial cells from the broth. The resulting culture was resuspended in 5% autoclaved distilled water, and the samples were subjected to heat treatment by incubating at 100 °C for 30 min [22]. After heat treatment, all samples were cooled to room temperature and stored at −80 °C until further use. To confirm the survival of the heat-killed (HK) *L. paracasei*, plate counting was performed.

### 2.3. Cell Culture

Caco-2 cells were cultured in DMEM supplemented with 10% FBS, 1% non-essential amino acids, and 100 U/mL penicillin–streptomycin was utilized. The cells were maintained in a humidified atmosphere at 37 °C with 5% CO_2_, following established protocols [24]. Prior to seeding, the cells were initially grown in 75 cm^2^ tissue culture flasks until they reached approximately 80% confluence. Next, they were seeded into a 12-well Transwell (Corning, Lindfield, Sydney, Australia) cell culture chamber at a density of 1 × 10^4^ cells/cm_2_. The cells were then cultured for 21 days to reach confluence and differentiation to obtain intestinal epithelial cells [25].

### 2.4. Cell Viability Assay

A Cell Counting Kit (CCK) 8 (Abcam, Cambridge, MA, USA) assay was used to assess the viability of Caco-2 cells [4]. Briefly, Caco-2 cells were seeded in 96-well plates and maintained at 37 °C under a humidified atmosphere of 95% air and 5% CO_2_ for 24 h. Following this, the cells were treated with LPS (100 μg/mL) and various concentrations of HK-LP (0.01–100 μg/mL) for 6, 24, 48, and 72 h. Next, a CCK8 solution was introduced to the wells, and cells were further incubated at 37 °C. The formazan produced was quantified using a microplate reader (Thermo Fisher Scientific, Waltham, MA, USA) at 490 nm. The experiment was performed in triplicate with duplicate readings for each condition, and the cell viability was expressed as a percentage of the untreated control cells.

### 2.5. Measurement of Membrane Permeability of Caco-2 Cell Monolayers

To evaluate the tightness and permeability of the Caco-2 cell monolayer model, FITC-dextran (4 kDa) was utilized as a paracellular transport marker. The transmembrane permeability of FITC-dextran indicates the tightness and permeability of the cell monolayer [26]. To investigate the effect of LPS (100 μg/mL) and HK-LP (1 μg/mL) on Caco-2 cell monolayers, the cells were treated with either a vehicle or the aforementioned substance for 24 h at 37 °C. The fluorescence intensity was quantified using a multifunctional microplate reader (Thermo Fisher Scientific, Waltham, MA, USA) with excitation/emission wavelengths of 490/520 nm.

### 2.6. Trans-Epithelial Electrical Resistance (TEER) Assay

The TEER assay was performed according to the method of Ottman with modifications [27]. To set up the experiment, cluster plates with wells were used. The wells contained an outer medium (0.6 mL, basolateral side) and an inner medium (0.4 mL, apical side), and were seeded with 1 × 10^5^ cells/cm^2^. When TEER values exceed 300 Ω/cm^2^, 100 μg/mL of LPS was added to the apical side, and 1 μg/mL of HK-LP was introduced to the wells. After 24 h, the TEER values were measured using a Millicell ERS Voltometer (Millipore, Burlington, MA, USA).

### 2.7. Measurement of the Inflammatory Maker

To assess the effects of LPS and HK-LP on Caco-2 cell monolayers, cells were treated with either a vehicle or 100 μg/mL of LPS, with or without 1 μg/mL of HK-LP, for varying lengths of time. After incubation for 24 h at 37 °C, the supernatant was collected via centrifugation at 8000× *g* for 20 min, and the levels of inflammatory markers were determined using an ELISA kit from Proteintech (Wuhan, China), following the manufacturer’s instructions.

### 2.8. Quantitative Real-Time Polymerase Chain Reaction (RT-qPCR) Analysis

The ABI Prism 7300 Real-Time PCR System (ABI, Wilmington, NC, USA) was used to detect the expressions of inflammatory factors and tight junctions in Caco-2 cells. To ensure accuracy and reliability, the relative expression of GAPDH mRNA was used as an internal reference. To calculate the relative gene expression, the 2^−△△Ct^ value was calculated [28]. The specific primer sequences used for the PCR analysis are listed in Table 1 [29,30,31,32,33].

### 2.9. Western Blot Analysis

To evaluate HK-LP repair at the protein expression level, a Western blot analysis was performed [34]. Caco-2 cell monolayers were exposed to either the vehicle or 100 μg/mL of LPS, in the presence or absence of 1 μg/mL of HK-LP. Following treatment, cells were harvested and homogenized as previously described [35], and their protein concentration was determined using a BCA kit (Solarbio, Beijing, China). Following this, equal amounts of protein were then separated by 7%–10% (*w*/*v*) sodium dodecyl sulfate–polyacrylamide gel electrophoresis and transferred onto polyvinylidene fluoride (PVDF) membranes (Millipore, Burlington, MA, USA). The membranes were blocked in 5% (*w*/*v*) bovine serum albumin (BSA) for 1 h at room temperature and then incubated overnight with primary antibodies and diluted in tris-buffered saline-Tween (TBST) at a 1:1000 dilution. After three washes in TBST, they were incubated with a HRP-conjugated secondary antibody (1:4000, Cell Signaling Technology) for 1 h at room temperature. The protein bands were visualized using chemiluminescence, and the relative amounts of proteins were quantified using ImageJ software.

### 2.10. Immunofluorescence Analysis

Caco-2 cells were treated with either the vehicle or 100 μg/ml of LPS, in the presence or absence of HK-LP (1 μg/ml). To prepare the cell samples for analysis, they were fixed with 4% paraformaldehyde at room temperature for 30 min, followed by permeabilization with 0.5% Triton X-100 diluted in PBS [36]. After blocking with 2% (*w*/*v*) BSA for 1 h, the samples were incubated overnight at 4 °C with rabbit anti-ZO-1 (Proteintech, Wuhan, China) and NF-κB p65 antibody. The cells were then washed three times in PBS and incubated with a diluted CoraLite488 fluorescent secondary antibody (1:500, Proteintech, Wuhan, China) for 1 h at room temperature. To visualize cell nuclei, DAPI staining was applied for 10 min. The samples were observed using a fluorescence microscope at 10× magnification (Leica, Wetzlar, Germany).

### 2.11. Data Statistics

The data were represented as the means ± standard errors of the means (SEMs) obtained from three independent experiments. Statistical analysis was performed using one-way ANOVA followed by DUNCAN’s multiple comparisons test in SPSS (version 17.0; IBM Corporation, Armonk, NY, USA). Lowercase letters (*p* < 0.05) were used to indicate significant differences between groups, with different letters representing different levels of significance.

## 3. Results

### 3.1. Effects of Nine HK-LP Strains on Pro-Inflammatory Factors Content

**Figure 1:**
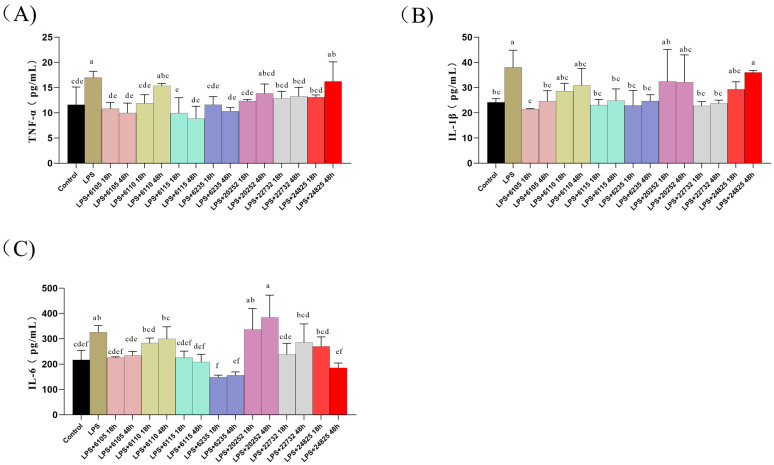
Effect of heat-killed *Lacticaseibacillus paracasei* (HK-LP) (1 μg/mL) at different cultivation times (18 h or 48 h) on TNF-α (**A**), IL-1β (**B**), and IL-6 (**C**) in lipopolysaccharide (LPS) (100 μg/mL)-treated Caco-2 cell monolayers after 24 h. HK-LP 6105, 6110, 6115, 6235, 20252, 22732, and 24825 were incubated for 18 h and 48 h, respectively; after that, they were heat-killed for 30 min at 100 °C. Control: untreated control cells; LPS: LPS-treated cells. Each result is represented as the mean ± standard error of the mean (SEM) from three independent experiments. Different lowercase letters (a, b, c, d, e, f) indicate different levels of significance (*p* < 0.05) and the same letters represent no significant difference (*p* > 0.05).

The current study aimed to investigate the effects of HK-LP on pro-inflammatory cytokines (IL-1β, IL-6, and TNF-α) in Caco-2 cell monolayers. ELISA was used to assess the cytokine levels after treating the cells with 1 μg/mL of HK-LP for 24 h (Figure 1). Our findings demonstrated that while HK-LP 6110, 20252, and 24825 did not significantly affect TNF-α content, other strains led to a significant decrease in TNF-α content compared to the LPS group (*p* < 0.05). Similarly, the IL-1β content in HK-LP 6105, 6115, 6235, and 22732 was significantly lower than in the LPS group (*p* < 0.05). Moreover, the IL-6 level was significantly decreased in HK-LP 6105, 6115, and 6235 after 18 h and 48 h of incubation compared to the LPS group (*p* < 0.05). Based on these findings, we selected HK-LP 6105, 6115, and 6235 for further experiments.

### 3.2. Effects of HK-LP on Caco-2 Cell Viability

The CCK8 assay was employed to determine cell proliferation and viability, revealing that both were influenced by the strain and culture time (Supplementary Figure S1). Notably, the lowest concentration (0.01 μg/mL) of HK-LP did not impact cell viability, regardless of strains or treatment duration. However, as the concentration of HK-LP and treatment time increased, the viability of the Caco-2 cells decreased. Specifically, exposure to 1.0 μg/mL of HK-LP for 24 h did not significantly reduce cell viability (*p* > 0.05). Nevertheless, higher concentrations of HK-LP (10 and 100 μg/mL) markedly reduced cell numbers after 24 h of treatment (*p* < 0.05). The cell viability assay also revealed that Caco-2 cells were more vulnerable to HK-LP 6105 and 6115 than to HK-LP 6235, as evidenced by the greater decrease in cell numbers observed following treatment with higher HK-LP concentrations (10.0 and 100.0 μg/mL) for a prolonged duration (≥24 h). Additionally, after 48 h of culturing, the Caco-2 cells were more sensitive to HK-LP than those with 18 h of culturing. Based on these findings, subsequent experiments employed a treatment of 1.0 μg/mL of HK-LP for 24 h to treat Caco-2 cells. 

### 3.3. HK-LP Treatment Restored LPS-Induced Damage in Caco-2 Cells Monolayers

After treatment with LPS (100 μg/mL), the integrity of the intestinal epithelial barrier in Caco-2 cell monolayers was disrupted, as indicated by a >20% decrease in the TEER value compared to the control group (71.7 ± 1.96%). Cells treated with LPS (100 μg/mL) were used in subsequent experiments. Treatment with 1.0 μg/mL of HK-LP resulted in a significant increase in the TEER value compared to the LPS group (*p* < 0.05). Interestingly, HK-LP 6235 demonstrated a more significant decrease in TEER caused by LPS after 18 h of culturing (88.99 ± 1.08%) compared to 48 h of culturing (94.34 ± 1.48%) (*p* < 0.05). 

**Figure 2:**
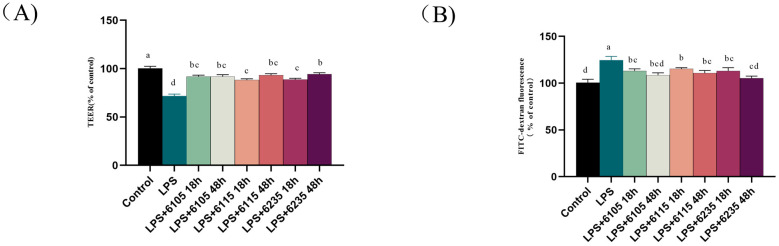
Effect of heat-killed L. paracasei (HK-LP) 6105, 6115, and 6235 (1 μg/mL) at different cultivation times (18 h or 48 h) on transepithelial electrical resistance (TEER) (**A**)and fluorescein isothiocyanate (FITC)-dextran fluorescence values (**B**) in lipopolysaccharide (LPS) (100 μg/mL)-treated Caco-2 cell monolayers after 24 h. HK-LP 6105, 6115, and 6235 were incubated for 18 h and 48 h, respectively; after that, they were heat-killed for 30 min at 100 °C. Control: untreated control cells; LPS: LPS-treated cells. Each result is represented as the mean ± standard error of the mean (SEM) from three independent experiments. Different lowercase letters (a, b, c, d) indicate different levels of significance (*p* < 0.05) and the same letters represent no significant difference (*p* > 0.05).

Confirming the barrier function of the Caco-2 cell monolayers paracellular transport of FITC-dextran (Figure 2B), LPS (124.55 ± 3.98%) (100 μg/mL) significantly increased FITC-dextran fluorescence compared to the untreated cells (*p* < 0.05). However, treatment with HK-LP resulted in a significantly lower FITC-dextran fluorescence value compared to the LPS group (*p* < 0.05). After 48 h of culturing, HK-LP 6105 (113.22 ± 2.04%) and 6235 (113.16 ± 3.43%) reduced the fluorescence value to the level of the untreated group, indicating that HK-LP repaired the LPS-induced increase in the permeabilization of the Caco-2 cell monolayer.

### 3.4. HK-LP Impacted LPS-Induced Inflammatory Factors in LPS-Induced Caco-2 Cell Monolayers

**Figure 3:**
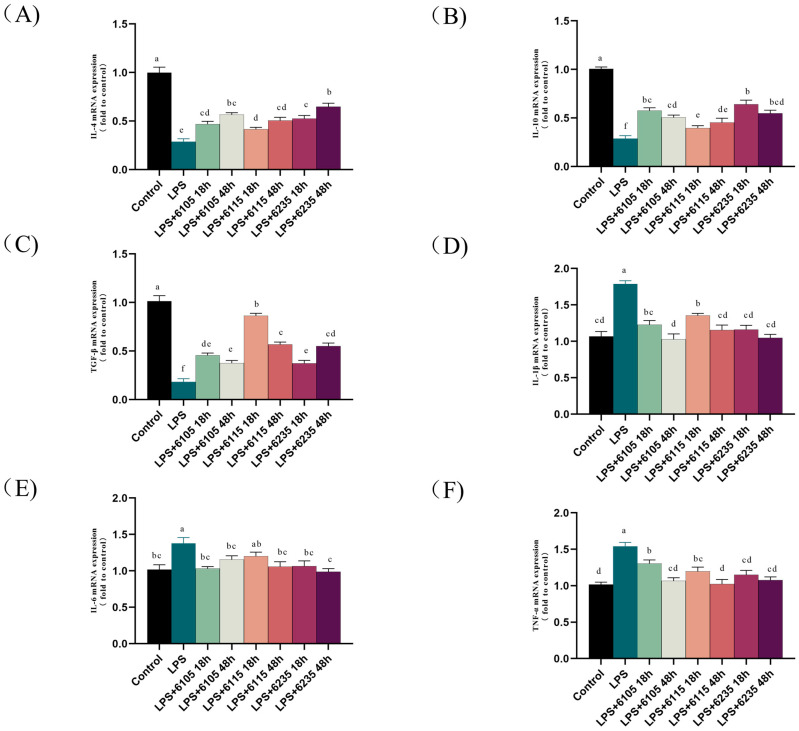
Interleukin (IL)-4 (**A**), IL-10 (**B**), transforming growth factor beta (TGF-β) (**C**), IL-1β (**D**), IL-6 (**E**), and TNF-α (**F**) mRNA levels in lipopolysaccharide (LPS) (100 μg/mL)-induced Caco-2 cell monolayers treated with heat-killed L. paracasei (HK-LP) (1 μg/mL). HK-LP 6105, 6115, and 6235 were incubated for 18 h and 48 h, respectively; after that, they were heat-killed for 30 min at 100 °C. Control: untreated control cells; LPS: LPS-treated cells. Each result is represented as the mean ± standard error of the mean (SEM) from three independent experiments. Different lowercase letters (a, b, c, d, e, f) indicate different levels of significance (*p* < 0.05) and the same letters represent no significant difference (*p* > 0.05).

RT-qPCR was utilized to measure the mRNA expression of several cytokines includingIL-1β, IL-6, TNF-, IL-4, IL-10, and TGF-β in LPS-induced Caco-2 cell monolayers. (Figure 3). LPS stimulation significantly increased the transcriptional levels of anti-inflammatory cytokines (*p* < 0.05), which were increased by supplementation with HK-LP. Notably, the mRNA expression of IL-4 was significantly lower in HK-LP 6235 after 18 h of culturing than after 48 h incubated (*p* < 0.05). Additionally, the TGF-ß mRNA expression in HK-LP 6115 was significantly higher with 18 h of culturing than after 48 h (*p* < 0.05). Conversely, the expression of pro-inflammatory cytokines was inhibited by LPS but upgraded by HK-LP. Specifically, in Figure 3F, TNF-α mRNA expression significantly differed between HK-LP 6105 and 6235 after 18 h and 48 h of incubation (*p* < 0.05).

### 3.5. HK-LP Changed the Expression Levels of Tight Junction Proteins 

**Figure 4:**
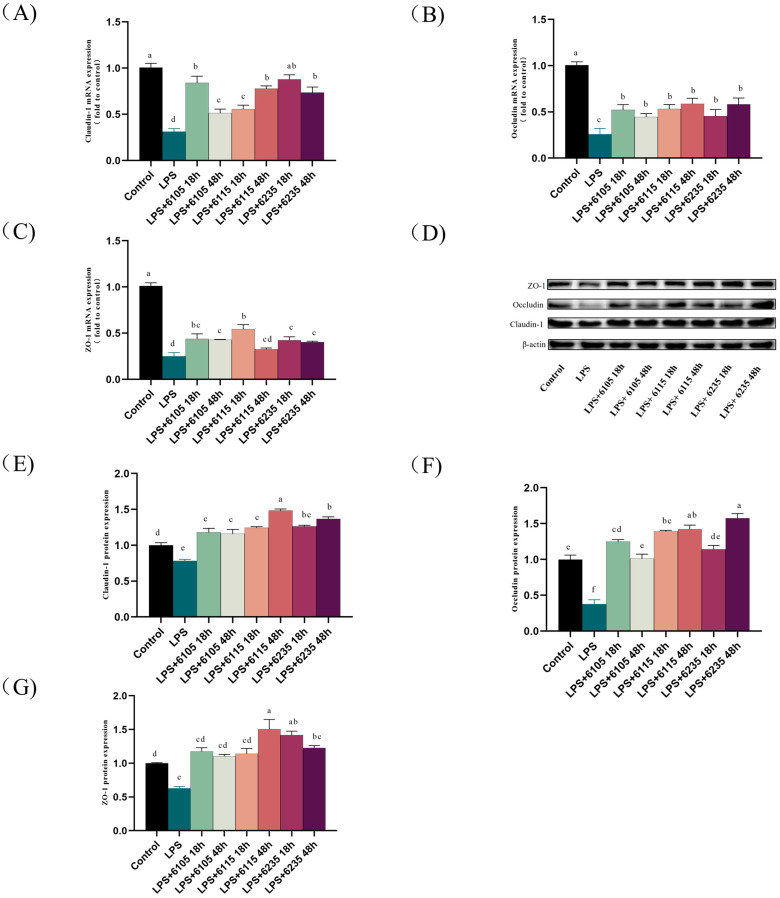

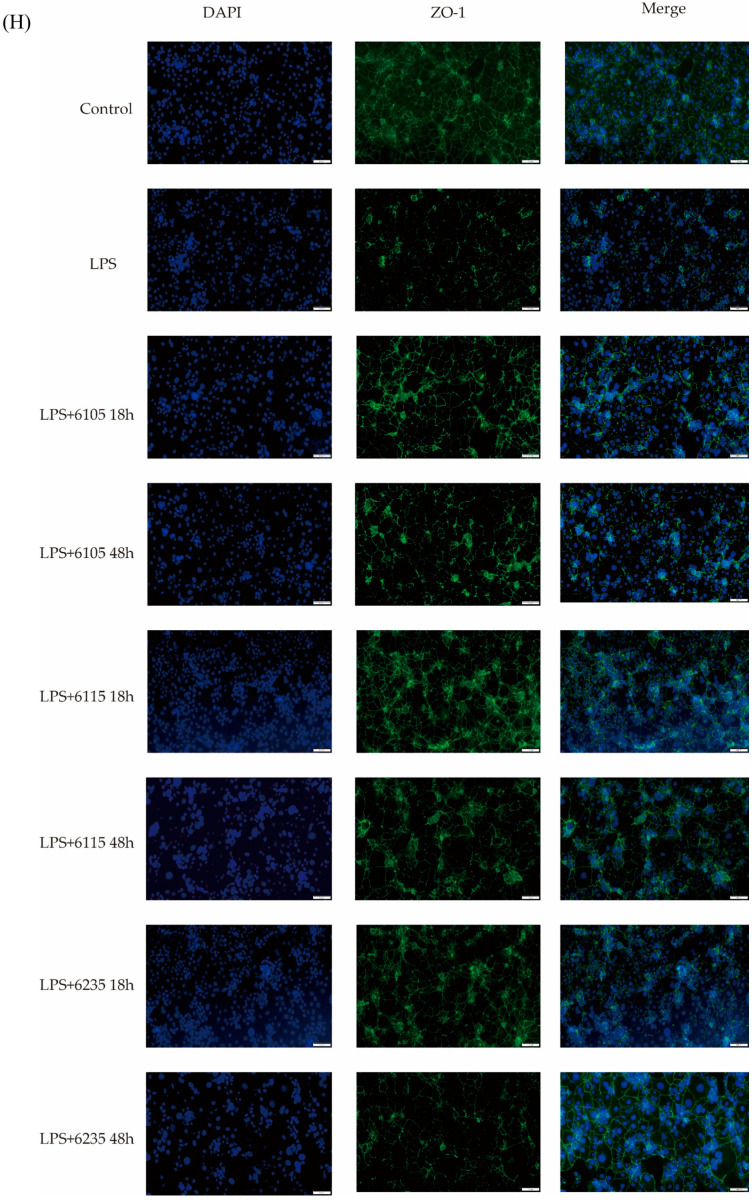
Effects of heat-killed *L. paracasei* (HK-LP) 6105, 6115, and 6235 (1 μg/mL) at different cultivation times (18 h or 48 h) on ZO-1 (**A**), claudin-1 (**B**), and occludin (**C**) mRNA levels, with Western blotting images of ZO-1, claudin-1, and occludin protein expression (**D**). Relative protein expression of ZO-1 (**E**), occludin (**F**), and claudin-1 (**G**). Cellular distribution of ZO-1 (**H**) in lipopolysaccharide (LPS) (100 μg/mL)-induced Caco-2 cells monolayers. HK-LP 6105, 6115, and 6235 were incubated for 18 h and 48 h, respectively; after that, they were heat-killed for 30 min at 100 °C. Control: untreated control cells; LPS: LPS-treated cells. Each result is represented as the mean ± standard error of the mean (SEM) from three independent experiments. L Different lowercase letters (a, b, c, d, e, f) indicate different levels of significance (*p* < 0.05) and the same letters represent no significant difference (*p* > 0.05).

The mRNA expression levels of the TJ protein markers ZO-1, claudin-1, and occludin were detected using RT-qPCR (Figure 4A–C). LPS significantly reduced the expression of these markers in Caco-2 cell monolayers, but this reduction was significantly reversed by HK-LP supplementation (*p* < 0.05). The protein expression level of ZO-1, claudin-1, and occludin was evaluated using Western blotting. A significant inhibition of ZO-1, claudin-1, and occludin protein levels was observed in the LPS group (*p* < 0.05) (Figure 4D), while HK-LP treatment restored their expression (Figure 4E–G). These findings indicated that HK-LP at different cultivation times inhibited the LPS-induced decrease in intestinal epithelial TJ protein expression.

The distribution of ZO-1 in Caco-2 cell monolayers was observed using immunofluorescence staining (Figure 4H). Untreated Caco-2 cell monolayers showed intact TJ networks. When the cell monolayer injuries were induced by LPS, the regular junctional complex was destroyed, showing an irregular arrangement. Specifically, HK-LP at different cultivation times improved barrier damage in intestinal epithelial cells. 

### 3.6. HK-LP Inhibited LPS-Induced TLRs in LPS-Induced Caco-2 Cell Monolayers

**Figure 5:**
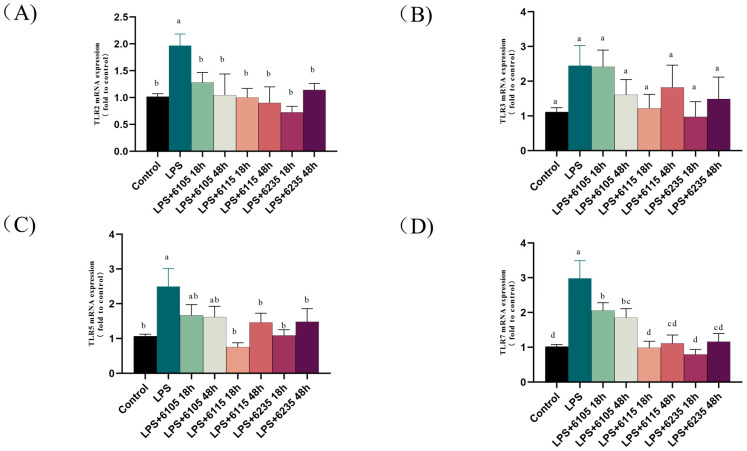
Effects of heat-killed *L. paracasei* (HK-LP) 6105, 6115, and 6235 (1 μg/mL) at different cultivation times (18 h or 48 h) on TLR2 (**A**), TLR3 (**B**), TLR5 (**C**), and TLR7 (**D**) mRNA levels in lipopolysaccharide (LPS) (100 μg/mL)-induced Caco-2 cell monolayers treated with heat-killed *L. paracasei* (HK-LP) (1 μg/mL). HK-LP 6105, 6115, and 6235 were incubated for 18 h and 48 h, respectively; after that, they were heat-killed for 30 min at 100 °C. Control: untreated control cells; LPS: LPS-treated cells. Each result is represented as the mean ± standard error of the mean (SEM) from three independent experiments. Different lowercase letters (a, b, c, d) indicate different levels of significance (*p* < 0.05) and the same letters represent no significant difference (*p* > 0.05).

The TLRs mRNA levels (TLR2, TLR3, TLR5, and TLR7) were detected via RT-qPCR (Figure 5). Our results show that TLR2 expression was significantly reduced in Caco-2 cells treated with HK-LP 6105, 6115, and 6235 for 18 h and 48 h, as compared to the LPS-treated group (*p* < 0.05). Additionally, TLR3 expression was not significantly influenced by HK-LP (*p* > 0.05). However, the expression of TLR5 in HK-LP 6115 and 6235 was significantly inhibited compared to the LPS-treated group (*p* < 0.05). Additionally, HK-LP 6105, 6115 and 6235 significantly downgraded the TLR7 expression compared to LPS-treated group (*p* < 0.05). Therefore, HK-LP 6105, 6115 and 6235 regulated the expression of TLR2, TLR5, and TLR7 in Caco-2 cells.

### 3.7. HK-LP Recovered TLR4/MyD88/NF-κB Signaling Pathway

**Figure 6:**
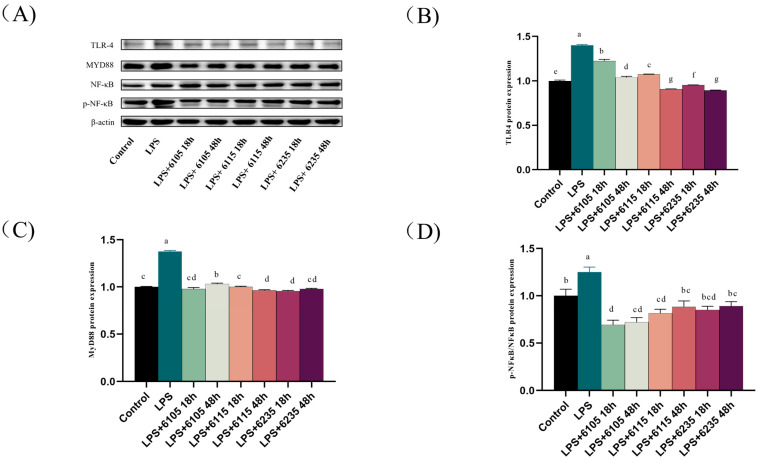

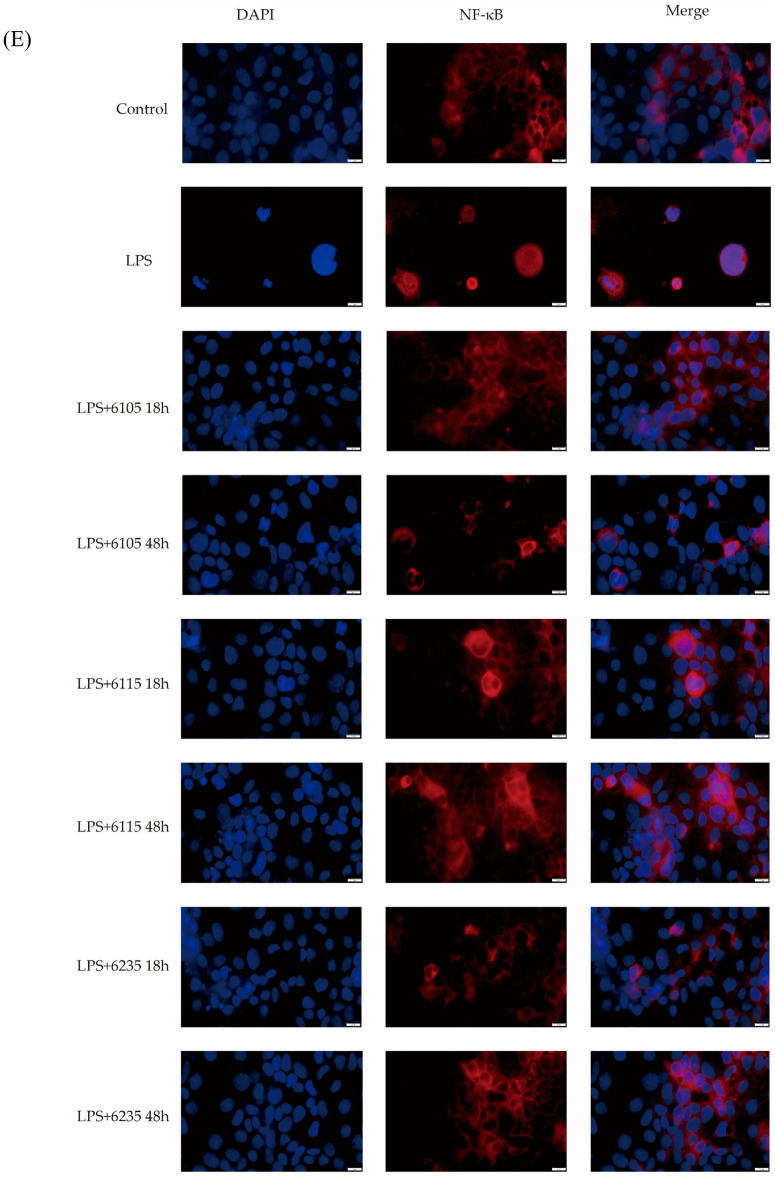
Effects of heat-killed L. paracasei (HK-LP) 6105, 6115, and 6235 (1 μg/mL) at different cultivation times (18 h or 48 h) on the expression of TLR4, MyD88, p-NF-κB, and NF-κB ratios in lipopolysaccharide (LPS) (100 μg/mL)-treated Caco-2 cell monolayers. Western blotting images of TLR4, MyD88, p-NF-κB, and NF-κB ratios (**A**). Relative protein expression of TLR4 (**B**), MyD88 (**C**), p-NF-κ, and NF-κB ratios (**D**), and NF-κB translocation (**E**). HK-LP 6105, 6115, and 6235 were incubated for 18 h and 48 h, respectively; after that, they were heat-killed for 30 min at 100 °C. Control: untreated control cells; LPS: LPS-treated cells. Each result is represented as the mean ± standard error of the mean (SEM) from three independent experiments. Different lowercase letters (a, b, c, d, e, f, g) indicate different levels of significance (*p* < 0.05) and the same letters represent no significant difference (*p* > 0.05).

To investigate the impact of HK-LP on the improvement of Caco-2 cell monolayers induced by LPS, we examined the expression levels of inflammatory factors using Western blotting. The results showed that LPS treatment increased TLR4 and MyD88 expression; in contrast, HK-LP treatment decreased the expression of both TLR4 and MyD88 (Figure 6B,C). In addition, HK-LP downregulated NF-κB phosphorylation expression and prevented the translocation of the NF-κB subunit (Figure 6D,E).

### 3.8. HK-LP Improved the Modulation of the MLCK/MLC Pathway

**Figure 7:**
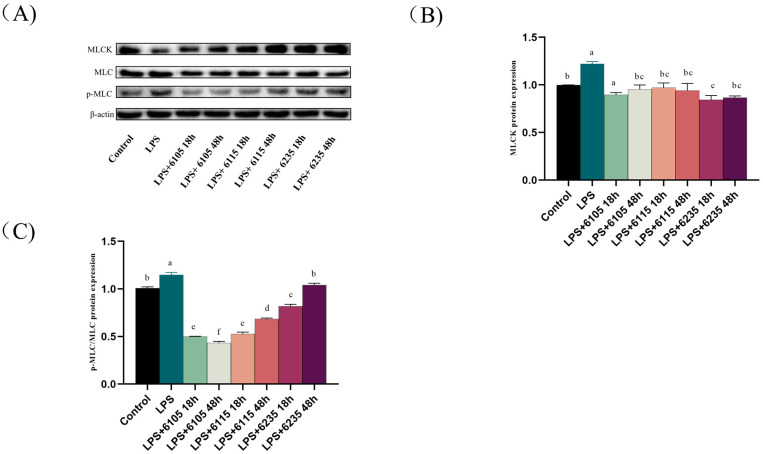
Effects of heat-killed L. paracasei (HK-LP) 6105, 6115, and 6235 (1 μg/mL) at different cultivation times (18 h or 48 h) on the expression of the myosin light-chain kinase (MLCK), p-myosin light-chain (MLC), and MLC ratios in lipopolysaccharide (LPS) (100 μg/mL)-treated Caco-2 cell monolayers. Western blot images of MLCK, p-MLC, and MLC ratios (**A**). Relative protein expression of MLCK (**B**), p-MLC, and MLCK ratios (**C**). HK-LP 6105, 6115, and 6235 were incubated for 18 h and 48 h, respectively; after that, they were heat-killed for 30 min at 100 °C. Control: untreated control cell; LPS: LPS-treated cells. Each result is represented as the mean ± standard error of the mean (SEM) from three independent experiments. Different lowercase letters (a, b, c, d, e, f) indicate different levels of significance (*p* < 0.05) and the same letters represent no significant difference (*p* > 0.05).

To investigate the regulatory mechanism of HK-LP in Caco-2 cell monolayers, we performed Western blotting to determine the levels of MLCK, MLC, and p-MLC proteins. LPS induced the upregulation of MLCK and MLC expression, whereas HK-LP inhibited these protein expressions (Figure 7). Our data demonstrated that HK-LP at different cultivation times inhibited the NF-κB nuclear translocation, thereby downregulating MLCK/MLC expression and safeguarding TJ proteins and the integrity of barriers.

## 4. Discussion

Intestinal epithelial barrier disruption is closely associated with dysfunction and various diseases. According to recent studies, the development of many metabolic diseases is triggered by the entry of various toxic chemicals and pathogens into the circulation, which is caused by a disturbed intestinal barrier [37]. Therefore, improving the intestinal epithelial barrier dysfunction is important for the treatment of these diseases. Many studies have supported that treatment with *L. paracasei* can increase the expression of tight junction proteins and control intestinal permeability [38]. Recently, postbiotics have increasingly been seen as microorganisms and/or their parts that help the host’s health, having beneficial biological activities for the prevention of metabolic diseases [39]. Experimental studies show that postbiotics alleviated the effects of metabolic diseases [40]. Therefore, we screened and examined the effects of heat-killed *L. paracasei* strains with different cultivation times on the function of the intestinal barrier and explored the expression of tight proteins and inflammatory factors by shielding the monolayer of Caco-2 cells against LPS-induced Caco-2 cell monolayers.

We used an LPS-induced Caco-2 cell monolayers model to assess the regulatory effects of *L. paracasei* strains with different cultivation times on intestinal barrier dysfunction. The human colon adenocarcinoma-derived Caco-2 cell lines have a microvillous shape that allows them to produce small intestine brush border epithelium-associated enzymes [41]. After 21 days of culturing, the cell monolayers formed a barrier structure that resembled the morphology and function of the human small intestinal epithelium [42]. LPS, an important component of Gram-negative bacteria, can alter the permeability and electrical resistance of intestinal epithelial cells [43]. Therefore, Caco-2 cells were added to the Transwell culture plate to construct cell monolayers, and LPS was induced to establish a model of intestinal barrier dysfunction. The TEER value and FITC-dextran paracellular transport were evaluated to determine the effects of LPS and HK-LP on the integrity of the intestinal barrier (Figure 2) [44]. Previous studies indicate that heat-killed *L. paracasei* PS23 sustainably increased the TEER value of intestinal epithelial cells induced by DSS and enhanced the integrity of the intestinal barrier [45]. Additionally, *Lactiplantibacillus plantarum* did not affect the value of TEER and permeability but alleviated inflammation-induced barrier dysfunction in Caco-2 cell monolayers [46]. We speculate that the difference in permeability of Caco-2 cell monolayers is because of the different genus of HK-LP strains. Additionally, the cell composition diversity of HK-LP cultured at different times may affect intestinal barrier. For example, *Lactobacillus* exopolysaccharides are synthesized in large quantities during the late stage of fermentation when the nutrients to support growth are limited [47]. However, there is no evidence to suggest the relationship between heat-killed probiotics at different cultivation times and their health-promoting effects. Therefore, future studies are required to elucidate this possibility.

The gut epithelium is the crucial barrier that protects the body from external harmful chemicals; therefore, it is also an area that frequently experiences local inflammation [48]. Previous studies have shown that intestinal mucosal immunity can be activated when the intestinal barrier is compromised. However, inflammatory mediators such as cytokines and chemokines are released, which increase in the intestinal tract and flow into the circulatory system, potentially leading to systemic inflammation [49]. An imbalance in cytokine immunity has been observed in compromising the intestinal barrier, demonstrating a reduction in anti-inflammatory molecules and an increase in pro-inflammatory cytokines [50,51]. TNF-α, IL-1β, and IL-6 are three critical pro-inflammatory cytokines that are dysregulated during damage to the intestinal epithelial barrier or an onset of intestinal inflammation [52]. In addition, LPS can affect the permeability of intestinal epithelial cells and stimulate the expression of inflammatory factors in the intestine, leading to local or systemic inflammatory responses that further damage the intestinal barrier [53,54,55]. Hence, as shown in Figure 3, we observed the regulatory effect of HK-LP cultured at different times on the relative mRNA expression of inflammatory factors in LPS-induced Caco-2 cell monolayers. Our results revealed that HK-LP at different cultivation times significantly attenuated LPS-induced IL-1β, IL-6, and TNF-α, as well as increased the content of IL-4, IL-10, and TGF-β (*p* < 0.05). Our findings are in accordance with a previous study showing that heat-killed *B. lactis* BB-12 and LGG effectively inhibited the production of IL-6 and IL-18 [17]. 

TJ proteins, such as occludins, claudins, and ZO proteins, are critical for the formation and maintenance of junctional complexes. However, LPS has been shown to impair the ability of the TJ barrier to protect epithelia [56], leading to inflammation and increased intestinal permeability [57,58]. Our research indicates that LPS (100 μg/mL) decreases TJ protein expression at both mRNA and protein levels, which aligns with our previous publication [26]. The present study showed that HK-LP dramatically improved intestinal epithelial barrier homeostasis and reduced paracellular permeability. Notably, HK-LP treatment significantly increased the expression of occludin, ZO-1, and claudin-1 in LPS-challenged cells (Figure 4).

LPS is a well-known TLR4 ligand that induces inflammatory responses by activating the TLR4/NF-κB signaling pathway and stimulating the junctional protein, MyD88 [59]. NF-κB controls the release of various inflammatory factors and regulates host inflammation, immune response, and cell developmental characteristics by increasing the production of inflammatory mediators and immune-related genes [60]. The cytokines stimulated by NF-κB stimulates can be used as activators of the NF-κB pathway, causing a greater release of inflammatory factors, activating NF-κB further, creating a positive self-regulation loop, amplifying the inflammatory response, and lengthening the period of chronic inflammation [61]. It has been reported that the interaction between LPS and TLR4 intestinal permeability increased the inflammatory response, which is related to the pathogenesis of organismal diseases [62]. Furthermore, MyD88, a critical splice molecule of the TLR, is also an important bridging protein that mediates NF-κB activation and cytokine production [63]. As shown in Figure 5, HK-LP significantly reduced the mRNA levels of TLR2, TLR5, and TLR7. It was suggested that TLR2, TLR5, and TLR7, not TLR3, may rely on the MyD88 signaling pathway, which activates NF-κB and initiates a signaling cascade that releases cytokines with inflammatory factors [64]. Previous studies have noted the importance of live and pasteurized Akkermansia in regulating intestinal barrier function [65]. As shown in Figure 6, LPS increased TLR4, MyD88, and NF-κB protein expression, and then HK-LP inhibited LPS-induced NF-κB and p-NF-κB protein expression. Combined with the present data, HK-LP consumption regulates the expression of inflammatory cytokines [66]. Thus, HK-LP at different cultivation times reduced LPS-induced expression of MyD88 and suppressed the TLR4/NF-κB signaling pathway.

MLCK is the major calmodulin kinase that regulates intestinal mucosal permeability. The MLCK signaling pathway regulates the cytoskeleton by mediating MLC phosphorylation [67]. MLCK was activated via LPS stimulation, and activated MLCK can cause phosphorylation of the MLC, mediate actin contraction, cause cytoskeleton remodeling, destroy cell TJ proteins, and thus increase the permeability of epithelial cells [68]. In addition, the nucleus receives the activated NF-B p65 subunit, which initiates MLCK transcription and increases MLCK protein production. Interestingly, LPS stimulation of intestinal epithelial cells simulates the production of inflammatory factors in vivo, further disrupting the intestinal epithelial barrier and activating the release of inflammatory factors [69]. Alternatively, the TJ protein expression is disrupted by MLCK protein activation, which disrupts TJ protein structure and function [70]. As shown in Figure 7, our results corroborate previous research and show that the overexpression of MLC protein mediates MLCK phosphorylation, which is required for intestinal epithelial barrier failure caused by LPS [71]. This observation indicates that MLC phosphorylation was activated by MLCK. Hence, our study demonstrated that HK-LP inhibits the activation of the MLCK/MLC signaling pathway and may potentially lead to the repair of the intestinal barrier in Caco-2 cell monolayers.

## 5. Conclusions

Our research demonstrated that HK-LP at different cultivation times regulated inflammatory cytokine production and upregulated TJ protein expression in LPS-induced Caco-2 cell monolayers. Furthermore, HK-LP ameliorated LPS-induced intestinal barrier dysfunction through the NF-κB/MLC signalng pathway. Thus, HK-LP has the potential to be used as a dietary supplement to prevent or slow the progression of intestinal disorders. In the future, we will elucidate HK-LP’s potential prevention mechanism of intestinal disorder progression in vivo. 

## Figures and Tables

**Table 1:** Primers of RT-qPCR.

Gene	Forward Primer	Reverse Primer
IL-4	TCTTTGCTGCCTCCAAGAACA	GTAGAACTGCCGGAGCACAG
IL-10	CGCTAGAACCAAGCTGTCCT	CACATGCGCCTTGATGTCTG
TGF-β	AGCAACAATTCCTGGCGATACCTC	TCAACCACTGCCGCACAACTC
IL-1β	TGACGGACCCCAAAAGATGA	TCTCCACAGCCACAATGAGT
IL-6	TGAAGCACCCACCAATACAA	CCAACCTCAGAAAGCAGCTT
TNF-α	CCCTCACACTCAGATCATCTTCT	CTACGACGTGGGCTACAG
iNos	GGAGCGAGTTGTGGATTG	CCAGGAAGTAGGTGAGGG
ZO-1	GGATGTTTATCGCATTGTA	AAGAGCCCAGTTTTCCATTGTA
Occludin	TCTAGGACGCAGCAGATTGG	TGGACTTTCAAGAGGCCTGG
Claudin	AGTTAGGAGCCTTGATGCCG	GCACAGGGAGTAGGATACGC
TLR2	CTTCACTCAGGAGCAGCAAGCA	ACACCAGTGCTGTCCTGTGACA
TLR3	GCGCTAAAAAGTGAAGAACTGGAT	GCTGGACATTGTTCAGAAAGAGG
TLR5	CCTTACAGCGAACCTCATCCAC	TCCACTACAGGAGGAGAAGCGA
TLR7	CTTTGGACCTCAGCCACAACCA	CGCAACTGGAAGGCATCTTGTAG
GAPDH	TGGAGAAACCTGCCAAGTATGA	TGGAAGAATGGGAGTTGCTGT

## Data Availability

Data have been described in this article.

## Supplementary Materials

The following supporting information can be downloaded at: https://www.mdpi.com/article/10.3390/nu15071758/s1, Figure S1: Viability of Caco-2 cells treated with heat-killed *L. paracasei* (HK-LP) (0, 0.01, 0.1, 1, 10, and 100 μg/mL) for 6, 24, 48, and 72 h.
