# Supplementary material for: Heat-Killed Lacticaseibacillus paracasei Repairs Lipopolysaccharide-Induced Intestinal Epithelial Barrier Damage via MLCK/MLC Pathway Activation
Source: Nutrients. 2023 Apr 4;15(7):1758. doi: 10.3390/nu15071758 (PMC10097264; doi:10.3390/nu15071758)
Supplement: Supplementary file 1 [file nutrients-15-01758-s001.zip › nutrients-2281300-supplementary.pdf]

Supplementary Material

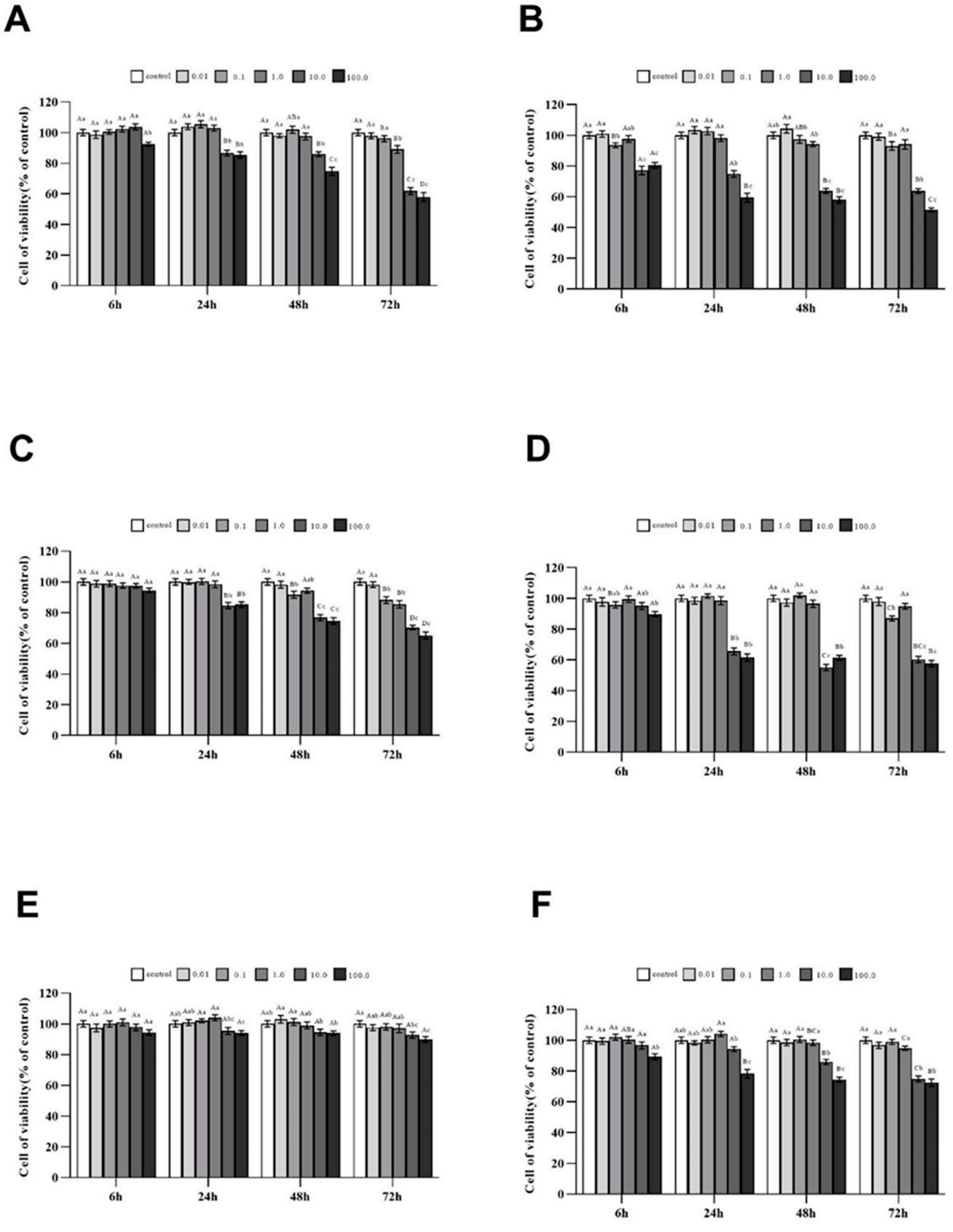

Supplementary Figure S1 Viability of Caco-2 cells treated with heat-killed *L. paracasei* (HK-LP) (0, 0.01, 0.1, 1, 10, and 100 μg/mL) for 6, 24, 48, and 72 h. The number of Caco-2 cells treated with HK-LP 6105 culture for 18 h (A) and 48 h (B), 6115 for 18 h (C) and 48 h (D), 6235 culture for 18 h (E), and 48 h (F). Each result is represented as the mean ± standard error of the mean (SEM) (n = 3). Different lowercase letters indicate significant differences between treatments with different concentrations of HK-LP at the same treatment time ($p < 0.05$). Different capital letters indicate significant differences between treatments with different time at the same HK-LP concentration ($p < 0.05$).
